# Strategy for Disease Diagnosis, Progression Prediction, Risk Group Stratification and Treatment—Case of COVID-19

**DOI:** 10.3389/fmed.2020.00294

**Published:** 2020-06-16

**Authors:** Mauno Vihinen

**Affiliations:** Department of Experimental Medical Science, BMC B13, Lund University, Lund, Sweden

**Keywords:** COVID-19, SARS-CoV-2, pathogenicity model, diagnosis, progression prediction, poikilosis

## Abstract

A novel strategy is presented for reliable diagnosis and progression prediction of diseases with special attention to COVID-19 pandemic. A plan is presented for how the model can be implemented worldwide in healthcare and how novel treatments and targets can be detected. The idea is based on poikilosis, pervasive heterogeneity, and variation at all levels, systems, and mechanisms. Poikilosis in diseases can be taken into account in pathogenicity model, which is based on distribution of three independent condition measures—extent, modulation, and severity. Pathogenicity model is a population or cohort-based description of disease components. Evidence-based thresholds can be applied to the pathogenicity model and used for diagnosis as well as for early detection of patients in risk of developing the most severe forms of the disease. Analysis of patients with differential course of disease can help in detecting biomarkers of diagnostic and prognostic significance. A practical and feasible plan is presented how the concepts can be implemented in practice. Collaboration of many actors, including the World Health Organization and national health authorities, will be essential for success.

## Introduction

**Figure 1:**
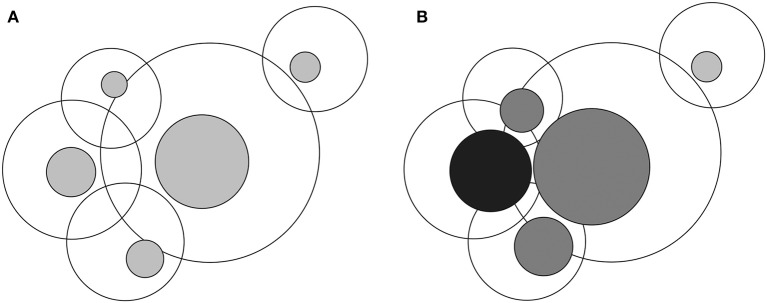
Visualization of interlinked levels and lagom and non-lagom variation. **(A)** In the normal situation heterogeneity within each level is of lagom (i.e., normal and acceptable) extent, indicated by gray zones inside the larger circles. Overlap of the circles indicates interactions of levels. **(B)** Once there is non-lagom extent of heterogeneity, black sphere, the extent, and location of the variation within the connected levels may be changed. The large circles depict all possible variations within each level and the colored circles the lagom variation zones. Multilevel effects arise due to extensive changes in levels that are highly connected and have different consequences, including diseases.

All biological systems are dynamic and show ubiquitous heterogeneity. A new concept, *poikilosis*, was recently introduced (Vihinen, submitted). It means inherent pervasive variation, heterogeneity, and fluctuation in living organisms, populations, ecosystems, and in their components and in processes within them. Each biological system, molecule, and process defines its own level within which fluctuations (i.e., heterogeneity) occur. Levels interact and can affect each other (Figure 1).

Despite poikilosis is pervasive, all variations, and their extents are not compatible and acceptable in biological processes and systems. Acceptable variation ranges are called for *lagom* and defined as suitable, sufficient, allowed, and tolerated extent of variation at any level in an organism, population, biological system, or process (Vihinen, submitted). Effects of non-lagom variations do not stay within the levels they emerge, they affect also interconnected levels. When variations are extensive, they cause diseases, and have multilevel effects first locally but can spread to become systemic. Poikilosis-based new definition for disease means: “a systemic deviation, defect or failure due to non-lagom variation leading to cumulative consequences in several levels.”

The extent of multilevel effects has wide personal range and further differences between individuals. When there are small variations, the system returns back to lagom level relatively quickly, and without major consequences. In the case of larger deviations, damage of some kind can occur, and impair or reduce the functionality, and adaptability of the system or organism. In the most severe conditions, a domino-like effect spreads to new levels and eventually causes death. The systemic extent in diseases displays wide heterogeneity between diseases and between individuals suffering from the same disease. According to the new definition, death is caused by excessive multilevel variations that irreversibly collapse vital processes and functions and spreads to become systemwide (Vihinen, submitted).

The concept of poikilosis can be implemented in practice. Here, a poikilosis-aware strategy is presented for COVID-19 due to SARS-CoV-2 pandemic. The principles are general and applicable to any disease.

## Concept of Pathogenicity Model

The new definitions for poikilosis, disease, and death can be implemented into practice in a pathogenicity model (PM) that describes the condition jointly by the combined effect of three factors—extent, modulation, and severity (1). These three independent constituent measures together describe the disease and indicate heterogeneity between the individuals as well as the continuum of phenotypes. The model can be used for several purposes including disease diagnosis, patient stratification, and prediction of disease progression.

PM is constructed based on the distributions of the constituent measures in a cohort of healthy and diseased individuals (1). Jointly, the three components define pathogenicity in all situations. According to the definition, severity of the disease indicates the stage, or degree to which a disease is expressed. Extent measures the breadth of disease appearance. Modulation summarizes the combined effect of factors that modify the disease phenotype.

The model is based on the definition of three measures that are specific for each disease, thus a dedicated PM is needed for every condition. Although complete PM implementation has been missing, there are already several approaches for determining the components of PM. Disease severity schemes and scoring systems have been developed [e.g., for type 1 Gaucher disease (2), follicular lymphoma (3), acute pancreatitis (4), sepsis-related organ failure assessment (5), and for staging, and grading of cancers (6), and other diseases].

The extent of disease has disease-specific definitions. For example, it can mean the spread of a tumor (7), affected surface area in Crohn's disease (8), or cutaneous T-cell lymphoma (9), or plaque distribution in coronary heart disease (10). There are some disease-specific extent indexes, such as in Wegener's granulomatosis (11) and coronary artery plaques (12–16).

Although important, the combined effects of modulators on phenotype have seldom been studied. Which factors are relevant depend on the condition. The modulators can include age, sex, ethnicity, body mass index, disease, and nutritional history, nutritional status, presence/absence of modifier molecules, complex genome-environmental interactions, immune system status, and history of infections, constitution of microbiota, and others. Genetic factors are important in many diseases and can include genetic variants, copy number variations (CNVs), *cis-* and *trans*-modifiers, allele dosage, imprinting, lyonization, overall expression regulation, and epigenetics, among many possible effectors. With relevant weights, even multimorbidities can be included to the modulation measure. Scores are already available for estimating the combined effects of some coexisting diseases, examples include the Charlson (17) and the Elixhauer indexes (18) and the Cumulative Illness Rating Scale (CIRS) (19).

**Figure 2:**
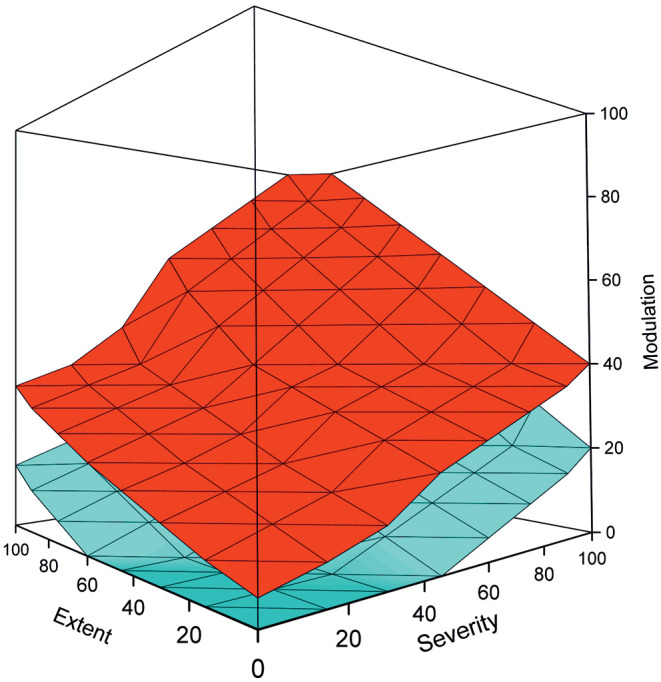
Example of a pathogenicity model, adapted from (1), shows the upper (red), and lower (cyan) boundaries for the pathogenicity zone. The space between these boundaries is filled by cases in the cohort. The shape, steepness, and other characteristics of the PZ depend on the disease. Benign cases are at the bottom of the graph, while the severely ill ones have high scores on all the three measures and are on the top of the figure. It is possible to apply various evidence-based thresholds to the PM for diagnosis and other purposes. By using temporal data and several data points per individual, the course of the disease can be followed. The model can be used also to stratify patient groups and to predict the course of disease and the outcome for individual patients.

PM is best visualized as a cube where the disease components are on the axes (Figure 2). The cube contains data for a cohort or a population. The data points form a cloud through the cube that ranges from the benign cases to the most severe condition within the disease. The range is always the same for all diseases, only that the severity can vary from relatively mild to life threatening. The cloud formed by the cases in the PM is called the pathogenicity zone (PZ). The shape, steepness, thickness and position of the PZ is characteristic for each disease.

## Pathogenicity Model for COVID-19

The number of diagnosed COVID-19 cases increases rapidly and more information is becoming available (20–22). For the PM a substantial number of cases is needed to cover the entire spectrum from benign to lethally ill. The required patient data is not publicly available and should thus be collected by the World Health Organization (WHO) and/or national health authorities. Once available, the model can be implemented and then applied worldwide.

Definitions of measures have to be agreed for extent, modulation and severity. One figure on the scale from minimum to maximum is required for each parameter per patient. These coordinates are then used to fill the PZ in the PM. Once an agreement is achieved on the definitions of the parameters, the data have to be harmonized as they are likely coming from numerous places and may have been obtained with somewhat different ways, different instruments etc.

What should be considered when defining the scores? In the case of extent, the disease can be local and range up to systemic state. Severity scale is from benign and symptomatic to the most severely ill with e.g., acute respiratory distress syndrome (ARDS), sepsis, or cardiovascular complications. Modulation is always a combination of several factors, in COVID-19 these include age, sex, tobacco smoking, hypertension, obesity, diabetes, and others (20–23). An evidence-based measure has to be devised to reflect the combined modulation effects. Once there is an agreement on the measures, the PM can be populated with cases.

## Diagnosis

Pathogenicity is a continuum ranging from benign and very mild cases to most severe, even lethal, forms. Based on known cases it will be possible to decide on a threshold (a plane or curve) that distinguishes in the PM the disease cases from healthy ones. This threshold can then be used for diagnosis of novel cases. The scores of the three measures differ for cases at the threshold, their combination provides strength for the diagnosis.

If necessary, the PM can be generated for different groups, in this case especially for age groups. Persons over 70 years old are at much higher risk than younger ones, thereby a dedicated PM, age correction or lower threshold in generic PM may be relevant for them. Once the PM is produced with sufficiently large population its application is very reliable to unknown cases. The PM can then be applied anywhere by determining the three scores. Computer programs can be devised to do it automatically from electronic health records.

## Stratification and Risk Groups

In addition to diagnosis, other evidence-based thresholds can be determined from the PM to identify subgroups of patients and individuals. This could be used for the stratification of patients for different purposes especially for early detection of those in increased risk. Many of the risk factors in COVID-19 are already known, but dedicated PM for the disease could facilitate even more reliable and early detection of patients, especially those in high risk for severe complications.

Subgroups can be detected based on known instances and analysis of their distribution within the PZ. Cases that cluster, (i.e., are closely located in the model), can be used for stratification. These clusters can then be used to define factors that are specific for them. Identified biomarkers can then be applied to diagnosis and risk assessment, and if necessary, also to redefine the measures in PM.

## Prediction of Disease Progression

The PM can be used also for further predictive purposes, especially by including temporal data for patients to follow the progression of their condition. In this case, pathogenicity scores are defined for patients during the course of disease and then connected into trajectories to indicate the progression of the disease. These trajectories differ in different parts of the PM and can be used to predict the course for novel cases once enough follow-up cases are included. This application could have a great impact for the early detection of patients who will need intensive care. In the case of COVID-19, follow-up data for diagnosis is needed just for a few days as the disease progression is so fast. Detection of risk cases as early as possible along with adequate treatment and follow-up will significantly contribute to the well-being of patients and help in directing the healthcare efforts in optimal way to follow the cases in the highest risk of severe complications, before having difficult to treat, and expensively treated systemic symptoms. Even extended longitudinal data will be beneficial for detecting long-term follow up and prediction of cases at the risk of harmful sequelae.

PM takes poikilosis, heterogeneity in the population, into account. This is essential as the clinical picture of patients varies greatly. PM distributes the cases into 3D space from where thresholds and clusters can be identified for diagnosis, stratification, and detection of patient groups for differential prognosis. This kind of stratification is much more reliable than simple classification based on biomarkers as the PM is based on population-wide heterogeneity and covers a range of factors in the constituent measures.

## Strategy for Developing Treatments

Numerous laboratories and companies are working to develop treatments for COVID-19. By considering poikilosis and PM a mechanism-based approach can be implemented. Severe COVID-19 leads to multimorbidity by affecting several bodily systems simultaneously, this is depicted in Figure 1. It is not possible to treat all the affected levels simultaneously in severe cases; however, by returning the systemic variations to lagom extent in some levels will affect also connected levels and reduce the total extent of non-lagom variations even substantially. Treatment of crucial actionable processes reduces the total burden of the disease. Detection and treatment of a small number of levels that are highly connected and thereby affecting many other levels should help to reconstitute more normal levels of heterogeneity. Apparently, more research is needed to detect all these interdependencies and the disease mechanisms.

An important factor in the treatment is to prevent body from entering to multilevel systemic state that can lead to collapse and eventual death. One central part of the treatment should be the utilization and activation of normal cellular and bodily systems that reduce, repair, or attenuate effects of harmful heterogeneity. Recently, TARAR countermeasures were introduced in relation to protein functional variations (Vihinen, submitted). TARAR means tolerance, avoidance, repair, attenuation, and resistance. Cells and organisms have numerous active and passive processes that restrict and limit the effects of all kinds of variations. Reconstitution and activation of these processes can be used to control and reduce the effects of diseases. Thus, systems biological understanding is needed for the entire progression of COVID-19 to detect normal mechanisms and processes that can be activated to fight against the disease. This can be achieved by pooling existing information about the disease, its symptoms and progression to information of affected levels and mechanism that can be used to enforce or trigger bodily countermeasures in addition to e.g., usage of medicines.

## Implementation of the Novel Strategy

The presented plans are feasible and can be implemented in multiple steps, many of them simultaneously. It would need collaboration between numerous actors to combine sufficient amount of information and cases, to develop the predictors, test them and to apply the system into practice in healthcare. For the latter, automatic systems can be developed to collect information from existing patient data.

The suggested steps are as follows:

### Development of Pathogenicity Model

- WHO or national authorities collect/provide health records and disease details for a substantial number of cases ranging from asymptomatic and mild disease cases all the way to the most severely ill patients. Relational database would be an optimal solution for storing and using these data.
  At the moment it is impossible to estimate how many cases are needed to populate the PM as it depends on so many factors. Preliminary calculations start from around 1,000 cases distributed throughout the PZ, with increased resolution achievable with additional cases.
- Agreement for how to define the measures—extent, modulation, and severity. Experts in the field have to agree on how these measures are obtained.
- It will be necessary to systematize and harmonize the parameters, laboratory measurements, and other clinical data. The clinical and other features may be defined and measured in different ways in different hospitals and in different countries. The included cases have to be defined in a single systematic way. Methods and computational tools can be developed to harmonize data from different sources.
- Once sufficient amount of data is available, the pathogenicity model can be constructed. It is possible to start with a smaller number of harmonized cases and update the model subsequently with additional cases. Once experience is gained from the use of PM it will be possible to find whether local or other adjustments are required.
- Definition of evidence-based thresholds for diagnosis, stratification, etc. Based on known cases these thresholds can be identified from the PM.
- It will be necessary to optimize the PM for deciding on the thresholds for diagnosis and other purposes.
- Systematic method testing with cases not included in the optimization steps. This stage provides information about the predictive performance of the PM.
- Now the method is ready to be distributed to hospitals and other healthcare units for diagnostic and stratifications purposes. To facilitate worldwide use, a web service with user-friendly interface or downloadable program that can be ported to existing electronic healthcare management systems has to be made.

### Steps for Implementing Disease Progression Prediction

- Collection and compilation of temporal data to generate a progression predictor. These data can be achieved for patients at hospitals; however, it would be important to have follow-up data also for patients with mild form of disease and even for asymptomatic individuals to cover the entire spectrum of disease progression courses.
- Analysis of the collected data to detect trajectories for different disease phenotypes and outcomes. Development of a tool to predict the course of disease. By analyzing the obtained strata biomarkers can be identified for more specific diagnosis.

### Identification of Actionable Processes and Countermeasures and Their Use for Treatment

- Identification of key systems and mechanisms affected by the disease. This will require holistic, systems biological approach to identify cellular and physiological processes affected by the disease. It is necessary to be able to understand how the virus infection impairs bodily functions.
- By knowing how the disease evolves and what mechanisms are involved it will be possible to identify actionable processes, particularly those which can be treated with existing regimes and therapies, preferably several affected levels simultaneously.
- Identification of the disease mechanism processes that can be treated by activating and enforcing known TARAR mechanisms.
- Investigating how the treatments of actionable processes and TARAR mechanisms reduce the burden of the disease.
- Development of guidelines for treatment modalities. International collaboration will be a key for success.
- Application of the PM, actionable treatments, and TARAR activation/modification processes to reconstitute multilevel systemic variation back to lagom or near-lagom levels to facilitate healing of patients.

## Summary

Here an approach and strategy are presented for how reliable diagnosis, prognosis, and stratification of patients can be achieved. Further, a systems biological scheme was presented for identifying processes and levels which can be treated with already available regimes, as well as a path to identify TARAR mechanisms, which can be activated, reinforced, or induced to reduce the effects and consequences of the disease. The scheme is feasible but does require joined forces to collect the medical information, development of the computational analysis, and prediction methods, as well as identification and application of the treatments. WHO is centrally placed for coordinating and collecting the required patient data and for the model implementation.

COVID-19 in the most severe, lethal, form is a systemic disease where processes, mechanisms, and molecules in multiple levels are simultaneously at non-acceptable, non-normal levels. This kind of complex multimorbidities are extremely difficult to treat. There is currently no curative treatment for COVID-19, apart from the body's own healing capacity, which is not sufficiently strong on many elderly and other risk group individuals. The approach presented here for combining medical treatments, activation of countermeasures, and PM as a predictive tool can be applied also to other diseases.

## Author Contributions

MV wrote the entire article and conducted the research.

## Conflict of Interest

The author declares that the research was conducted in the absence of any commercial or financial relationships that could be construed as a potential conflict of interest.
